# Disseminated tuberculosis presenting as irido-ciliary granuloma in an immunocompetent patient

**DOI:** 10.1007/s12348-012-0068-8

**Published:** 2012-03-28

**Authors:** Soumyava Basu, Ruchi Mittal, Suryasnata Rath, Praveen Kumar Balne, Savitri Sharma

**Affiliations:** 1 Retina-Vitreous Service, LV Prasad Eye Institute, Patia, Bhubaneswar, 751024 India; 2 LV Prasad Eye Institute, Patia, Bhubaneswar, 751024 India

Dear Editor,

The diagnosis of extra-pulmonary tuberculosis can be challenging, even in highly endemic countries. We report a case of irido-ciliary granuloma, where initial screening for TB was negative, but further investigations revealed multiple organ involvement with acid-fast bacillus (AFB), confirmed as *Mycobacterium tuberculosis* (MTB) by polymerase chain reaction (PCR).

## Case report

**Fig. 1:**
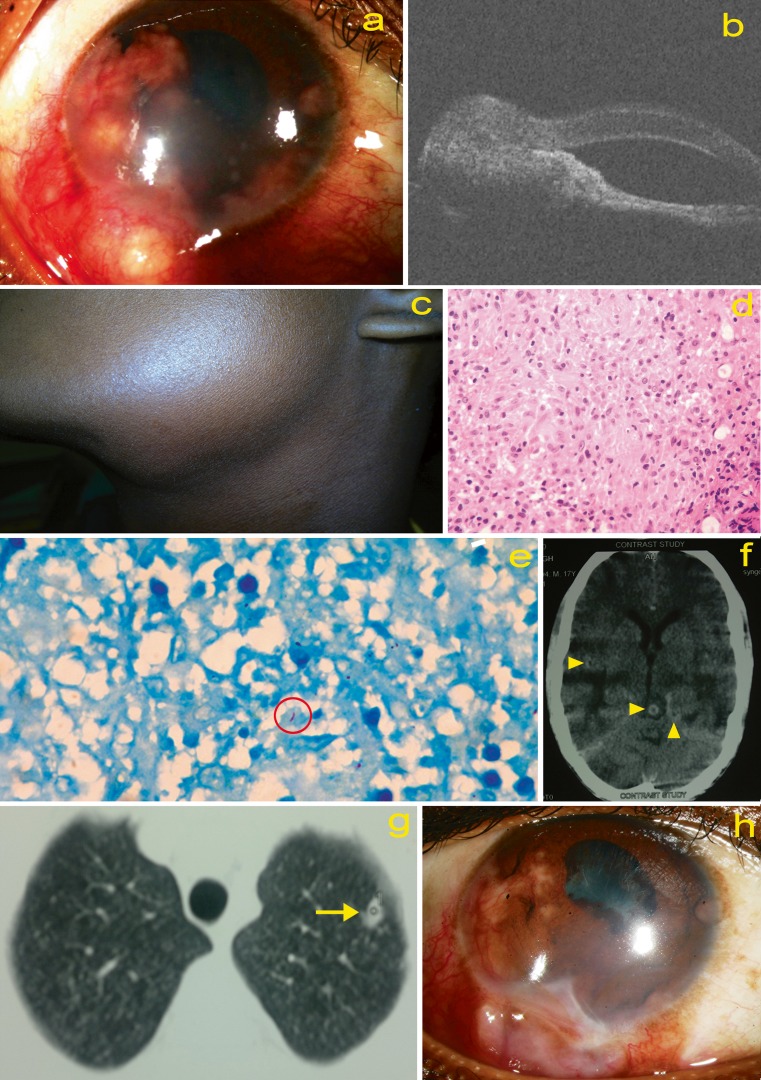
**a**, **h** Slit lamp photograph of the right eye showing multiple vascularized granulomas that seemed to arise from the angle of the anterior chamber and anterior surface of iris. A scleral nodule, partially extending into the cornea, is seen near the inferior limbus. However, there is no direct extension of the iris lesions into cornea. **b** Ultrasound biomicroscopy of right eye, showing continuation of the iris granuloma into the ciliary body and then onto the sclera. **c** Left submandibular lymphadenopathy. **d** Near confluent epithelioid cell granulomas in the sub-epithelium. Caseation necrosis within the tubercles is slight to absent (haematoxyin–eosin, ×400). **e** CT scan of the head (axial view) showing multiple ring enhancing lesions (*arrow heads*) in the brain parenchyma. **f** CT thorax showing small, non-cavitatory lesion (*arrow*) in the left apical lobe. **g** Slit lamp photograph 2 weeks post-treatment, showing partial resolution of iris granuloma and scleral nodule

A 17-year-old boy presented with reduced vision in the right eye for 2 months. Best corrected visual acuity (BCVA) was counting fingers 2 m and 6/6 in the right and left eyes, respectively. Intra-ocular pressures were in the right 18 mmHg and left 16 mmHg. Slit lamp examination of the right eye showed mutton fat keratic precipitates and multiple densely vascularized granulomatous lesions on the anterior surface of iris that seemed to arise from angle of anterior chamber (Fig. 1a). A vascularized scleral nodule, with surrounding ciliary congestion, was noted near the inferior limbus. The right fundus was not visible. The left eye showed optic disc edema, but no other inflammatory signs. B scan ultrasonography of the right eye showed disc edema, but not choroidal thickening or vitreous echoes. Ultrasound biomicroscopy of the right eye showed the iris lesion extending into ciliary body and then onto sclera (Fig. 1b).

Systemic examination revealed left submandibular lymphadenopathy (non-tender, matted, rubbery consistency; Fig. 1c). Based on the above findings, we diagnosed irido-ciliary granuloma with scleral extension in the right eye, associated with cervical lymphadenopathy of likely tubercular aetiology, and probable raised intracranial pressure. However, the tuberculin test was negative (4 mm induration with 5TU) and chest radiogram was normal. Fine needle aspiration cytology of the submandibular lymph node showed mixed population of reactive lymphoid cells with scattered histiocytes and plasma cells. There was no evidence of epithelioid granulomas or caseous necrotic material. Ziehl–Neelsen stain was negative for AFB.

We therefore biopsied the scleral nodule that revealed well-formed granulomas composed of epithelioid histiocytes, chronic lymphomononuclear cells and plasma cells and on 20 % acid-fast staining showed scattered AFB in the tissue (Fig. 1d). PCR showed positive for MTB with three different gene targets (IS6110, MPB64 and protein B).

Subsequently, computed tomography (CT) of head showed multiple ring enhancing lesions in the brain parenchyma (Fig. 1e). CT thorax showed a small non-cavitatory lesion in the left lung (apical lobe, Fig. 1f). Sputum tested positive for AFB. ELISA for HIV was negative. Based on neurologist’s recommendation, we initially treated the patient with intravenous dexamethasone (to reduce risk of paradoxical worsening of brain lesions) for 3 days, followed by five-drug ATT (anti-tubercular therapy—isoniazid, rifampicin, ethambutol, pyrazinamide and streptomycin) and oral corticosteroids (1 mg/kg/day, tapered). Irido-ciliary granuloma, optic disc edema and cervical lymphadenopathy gradually resolved over the next 2 months (Fig. 1g). BCVA of the right eye improved to 20/60. Thereafter, ATT was changed to isoniazid and rifampicin for another 7 months—right BCVA was 20/50, and iris lesions had completely resolved with minimal residual fibrosis.

## Comment

Disseminated TB refers to involvement of two or more non-contiguous sites and is commonly associated with immune-compromised state [1]. This case illustrates widespread dissemination of MTB (lung, eye, brain and lymph nodes) in an immunocompetent patient that presented initially with ocular manifestations. It also demonstrates the poor sensitivity of routine tests (in uveitis practice) like tuberculin test and chest radiography. Importantly, the tuberculin test has low positivity in disseminated TB [1], and pulmonary TB with normal chest radiograph is increasingly being documented [2]. In ocular TB (histo-pathologically proven) too, 40 % (7 out of 17) had negative tuberculin test, while 57 % (8 out of 14) had normal chest radiographs [3]. The advantage of obtaining relevant tissue/ body fluid for microbiological/histopathological evaluation in such situations is well highlighted in this case. Ocular TB, however, is extremely paucibacillary, and diligent search is required to identify the organism in the tissue [3]. The role of high-resolution chest CT over plain radiogram in patients with granulomatous uveitis, as described earlier [4], is also evident.

The risk of corneal/scleral involvement by iris/ciliary body tubercular granulomas has been previously described [5, 6]. Interestingly, the first description of uveal TB (Maitre-Jan, 1707) was apparently of an iris lesion perforating the cornea [4]. While the present case responded well to ATT, full-thickness eye wall resection may be required in non-responders [6].
